# Added value of radiological staging to clinical examination in different histopathological subtypes of uterine cervical cancer: A retrospective study

**DOI:** 10.1016/j.eurox.2025.100376

**Published:** 2025-03-06

**Authors:** Carla Linn Stadler, Sara N. Strandberg

**Affiliations:** a Hospital of Sundsvall, Sundsvall, Sweden; b University Hospital of Umeå, Department of Diagnostics and Intervention, Umeå University, Umeå, Sweden

**Keywords:** Uterine cervical neoplasms, Squamous Cell Neoplasms, Adenocarcinoma, Magnetic Resonance Imaging, Computed Tomography, Positron Emission Tomography Computed Tomography

## Abstract

**Objective:**

Accurate staging of uterine cervical cancer (UCC) is crucial for treatment guidance and prognostic predictions. This study investigated the added value of conventional diagnostic imaging for different histopathological subtypes of UCC by comparing clinical staging according to International Federation of Gynaecology and Obstetrics staging system (cFIGO) and radiological staging (rFIGO) with histopathological staging (pFIGO) as reference.

**Methods:**

26 consecutive patients with UCC from the retrospective part of the PRODIGYN study (ethical approval number 2022-04207-01; NCT05855941) were included in the present study. Data from study participants was collected from radiological and histopathological records 2016–2022 at the University hospital of Umeå. Staging was assessed according to the FIGO 2018 staging system. Statistical analysis included descriptive statistics and Cohen’s weighted kappa coefficient (κ) for calculation of agreement between cFIGO and rFIGO, and between rFIGO and pFIGO.

**Results:**

With rFIGO staging, more advanced disease stages were found in 67 % (8/12 patients with known cFIGO). Poor agreement was found between cFIGO and rFIGO (κ =0.057) and between rFIGO and pFIGO (κ= 0169). Among the patients with squamous cell carcinoma (SCC) positive for human papilloma virus (HPV+), 67 % (4/6) were assigned a higher stage by rFIGO compared to cFIGO. For the single patients with HPV-negative SCC and HPV status unknown SCC, both were upstaged by rFIGO. In the case of adenocarcinomas, 67 % (2/3) of the patients were assigned a higher stage with rFIGO.

**Conclusions:**

In primary staging of UCC, rFIGO leads to substantial up-staging compared to cFIGO, without obvious differences in subtypes.

## Highlights

• Radiological staging identifies higher disease stage compared to clinical staging.
• No staging differences between histopathological subtypes of Uterine Cervical Cancer.
• Poor agreement between clinical and radiological staging.
• Imaging detects lymph node and extra pelvic metastases compared to clinical exam.

## Graphical Abstract

**Figure:**
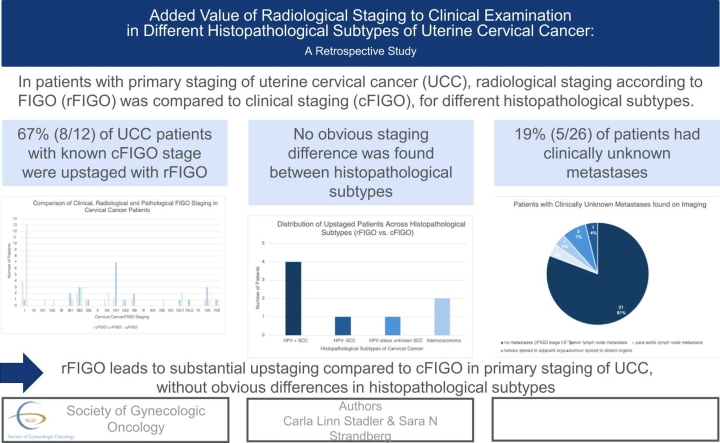

## 1 Introduction

Uterine cervical cancer (UCC) is the one of the most common cancers among women worldwide, with over 600,000 cases and 340,000 deaths annually [1]. It remains a significant challenge in gynaecological oncology, with accurate staging being essential for determining appropriate treatment modalities and prognostic outcomes [1], [2], [3], [4]. In Sweden, UCC ranks as the 10th most common cancer among women overall and rises to the third most prevalent among those aged 15–44 years [5].

Human Papilloma Virus (HPV) infection is the primary cause of UCC with over 100 identified genotypes divided into low-risk and high-risk strains. High-risk HPV types, particularly HPV 16 and HPV 18, are considered the most carcinogenic, with HPV 16 commonly associated with squamous cell carcinoma (SCC) and HPV 18 with adenocarcinoma [6]. The strong link between HPV infection and UCC underscores the need for effective diagnostic strategies to manage and treat this disease. With the introduction of HPV vaccination programs, which target these high-risk types, HPV-associated (HPV+) UCC is expected to decline in the future [7], [8]. This anticipated reduction in HPV+ UCC will shift the histopathological panorama, highlighting the importance of continuous validation of radiological disease staging for determining appropriate treatment strategy and accurate prediction of prognostic outcomes [1], [2], [3], [4].

Historically, the staging of UCC according to the International Federation of Gynaecology and Obstetrics (FIGO) classification predominantly relied on clinical examination, which may not accurately capture the full extent of tumour invasion [4], [9], [10]. Magnetic resonance imaging (MRI) has the potential to improve staging given its superior soft tissue contrast resolution which offers a detailed evaluation of tumour size, depth of stromal invasion, and lymph node involvement, while computed tomography (CT) and positron-emission tomography with CT (PET/CT) using 18F-fluoro-deoxy-glucose (FDG) render whole-body examinations [2], [4], [11], [12], [13], [14], [15]. Recognizing these aspects, FIGO was revised in 2018 with incorporation of imaging technologies to enhance staging accuracy [16], [17], [18], [19].

This retrospective study aims to systematically assess the added value of diagnostic imaging in primary staging of UCC for different histopathological subtypes, by comparing clinical staging (cFIGO) and radiological staging (rFIGO), with histopathological staging (pFIGO) as reference, according to the FIGO 2018 staging system.

## 2 Materials and methods

### 2.1 Patient selection and characteristics / inclusion & exclusion criteria

This retrospective study was part of the PRODIGYN study, approved by the Swedish Ethical Review Authority (ethical approval number 2022-04207-01; NCT05855941). Patient consent was waived. All procedures followed were in accordance with the ethical standards of the Helsinki Declaration.

Patients were eligible for the study if they met the following inclusion criteria: primary UCC presented at multidisciplinary gynaecological cancer conference, not previously treated, > 18 years old, and no other known malignancy within the last 10 years. Patients were excluded if imaging suggested another primary malignancy.

**Fig. 1:**
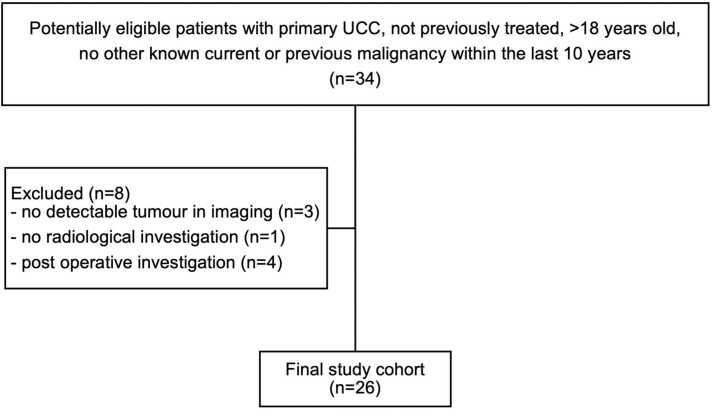
STARD Diagram of Patient Selection.

For this sub-study, an additional exclusion criterion was missing baseline pre-treatment cross-sectional imaging data (MRI, CT, and/or FDG-PET/CT). Ultimately, 26/34 patients were included as accounted for in the STARD diagram (Fig. 1). Data were collected from radiological and histopathological records between 2016 and 2022, at the University Hospital of Umeå.

**Fig. 2:**
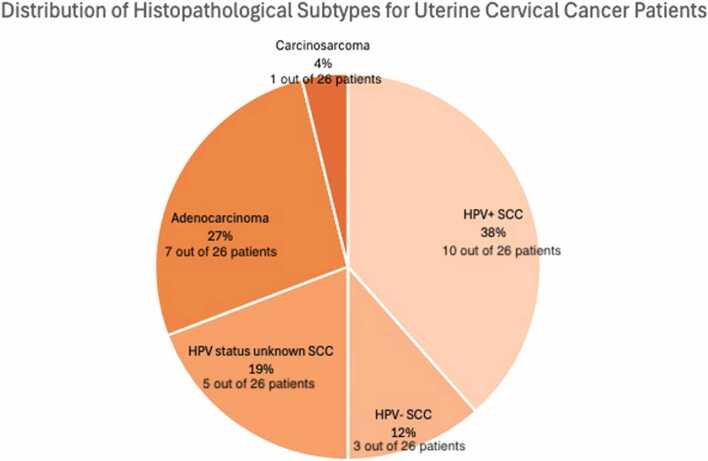
Distribution of Histopathological Subtypes for uterine cervical cancer (UCC) Patients: HPV-positive squamous cell carcinoma (HPV+ SCC), HPV-negative squamous cell carcinoma (HPV- SCC), HPV status unknown squamous cell carcinoma (HPV status unknown SCC), adenocarcinoma, and carcinosarcoma.

Fig. 2 illustrates the distribution of UCC histopathological subtypes within our cohort.

### 2.2 Imaging protocols

Imaging was performed according to clinical routine protocols, in the Region of Västerbotten, Sweden, during the years 2016–2022. 26 patients had an MRI, 22 had a CT and 4 had an FDG-PET/CT.

#### 2.2.1 MRI protocol

All studies were performed on 1,5 Tesla Signa Premier (GE Healthcare, Milwaukee, WI, USA) and 3 Tesla Discovery MR750 scanners (GE Healthcare, Milwaukee, WI, USA). A diagnostic MRI examination of the pelvis was performed in the feet first supine position using a body array coil. The following sequences were included: T2-weighted with and without fat suppression with a slice thickness of 3–4 mm, DWI (using b-values 0, 100, 800/1000) with a slice thickness of 4 mm. If feasible, contrast-enhanced MR images were obtained at baseline and 2.5–3 min after the intravenous administration of gadolinium chelate-based contrast (Dotarem 0.2 ml/kg) at a flow rate of 1–2 ml/s. For contrast-enhanced imaging, transverse and sagittal pre- and post T1 Dixon and/or Wave sequences were performed with a slice thickness of 1 mm.

#### 2.2.2 CT protocol

Preoperative CT scans were performed on Siemens Somatom Definition Flash 128-slice scanner (Siemens Healthineers, Forchheim, Germany) and GE Lightspeed VCT 128-slice and GE Revolution CT 256-slice CT scanners (GE Healthcare, Milwaukee, WI, USA). Standard thoraco-abdominal CT protocol included oral administration of 1000 ml of water 30 min prior to examination and intravenous bolus injection of low-osmolar contrast medium - Omnipaque 350 mg I/ml according to weight with maximum 80 kg for female and 100 kg for male, diluted in 30 ml NaCl. Images were obtained in the supine position from apex to crista in the arterial phase and from diaphragm to trochanter minor in the venous phase with scan parameters of 120 kV (or individualized kV on Siemens Somatom Definition Flash, CARE kV), 80–740 mA and 0.625 mm slice thickness.

#### 2.2.3 FDG-PET/CT protocol

Image acquisition was performed after 6 h of fasting, with blood glucose < 11 mmol/l, using a Discovery 690 PET/CT scanner (GE Healthcare, Milwaukee, WI, USA). A low-dose 120 kV 30 mA CT was first acquired for attenuation correction. Then the PET acquisition started 60 min post-injection of 4 MBq/kg FDG, with 2 min per bed position in time-of-flight mode. PET data was reconstructed using the iterative VuePoint FX, with 2 iterations, 24 subsets, 6.4 mm post-filter, with attenuation, scatter and decay corrections. Secondly, a contrast- enhanced CT was performed after a split-bolus intravenous injection of Omnipaque 350 mg I/ml, 0.5 g I/kg with 120 kV, 150–700 mAs Auto-mA (35 Noise Index) and 0.625 mm slice thickness. All examinations were performed in supine position with arms above the head, covering a field-of-view from the orbitomeatal plane to the proximal thighs.

### 2.3 Staging assessment

UCC staging was performed according to the FIGO 2018 classification. Assessment of cFIGO was based on findings from physical and clinical pelvic examinations conducted under general anaesthesia (EUA). Assessment of rFIGO included all available baseline CT, FDG-PET/CT and pelvic MRI scans. All staging information was retrospectively obtained from medical records, blinded to histopathological diagnosis.

### 2.4 Re-evaluation procedure

In two cases the radiological report was inconclusive, and a reassessment was conducted, blinded to histopathological diagnosis, as in the original reports. This was carried out by a radiologist and nuclear medicine physician with > 10 years’ experience in gynaecological-oncological imaging.

### 2.5 Statistical analysis

Descriptive statistics were calculated for the age, weight and height of the patient cohort. (Table 1)

**Table 1:** Descriptive statistics of age, weight and height.

Descriptive Statistics	Total Number of Patients	Minimum	Maximum	Mean	Standard Deviation
Age	9	40	84	58,89	18,917
Weight	21	40	117	78,62	16,500
Height	21	148	175	164,43	7507

The added value of rFIGO for the entire cohort was defined and described as frequency of metastatic disease.

The comparison and level of agreement between cFIGO and rFIGO, as well as rFIGO and pFIGO, were assessed using descriptive statistics and Cohen’s weighted kappa coefficient (κ). Reliability Statistics were calculated using Cronbach's Alpha for testing of internal consistency of the data, and Two-Way Mixed Intraclass Correlation Coefficient (ICC) for reliability.

Descriptive statistics (frequencies) were used for investigation of trends in different histopathological subgroups.

All statistical analyses were performed using SPSS software versions 28 and 29 IBM Corp. (Armonk, NY, USA), with a significance level of < 0,05.

## 3 Results

### 3.1 Descriptive statistics

Patients' characteristics are shown in Table 1.

### 3.2 Clinical vs. radiological FIGO staging

**Fig. 3:**
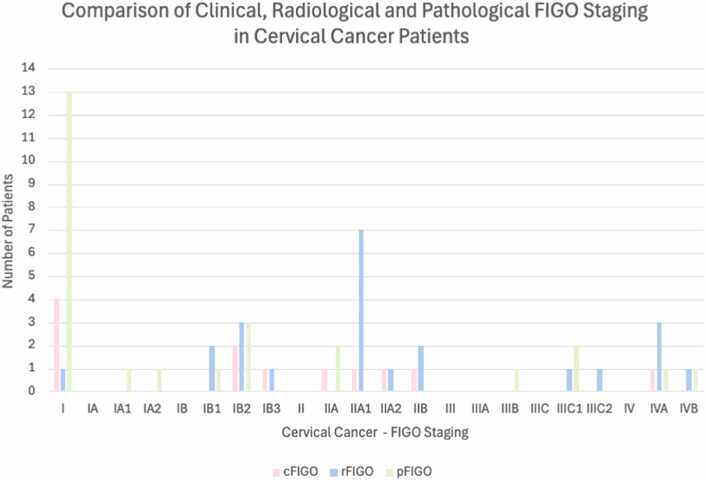
Comparison of clinical (cFIGO), radiological (rFIGO) and patholgoical (pFIGO) FIGO Staging. This bar graph compares the number of UCC patients categorized by each clinical (cFIGO – pink bars), radiological (rFIGO – blue bars) and pathological (pFIGO – green bars) FIGO stage. This graph highlights discrepancies and similarities between the three staging methods, showing variations in patient counts for each stage.

Fig. 3 illustrates a comparison between cFIGO (12/26), rFIGO (23/26) and pFIGO (26/26) staging for all included patients.

**Fig. 4:**
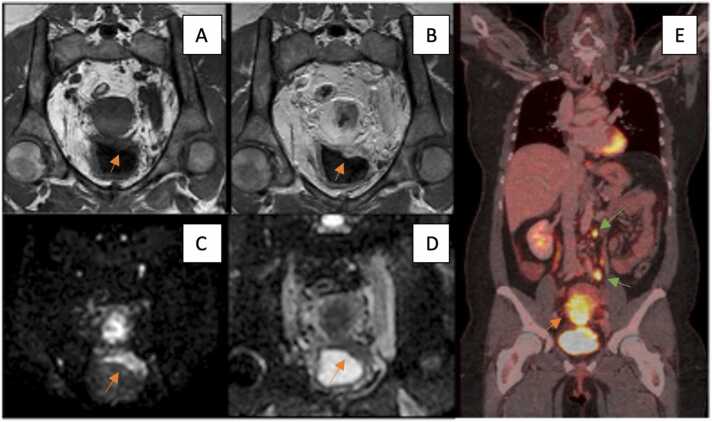
Cervical Carcinoma, MRI and PET/CT imaging. The orange arrow in Picture A (T1W TFE, coronal MRI) shows invasion of the tumour into the posterior wall of the bladder, with the orange arrow in Picture B (Gd T1W TFE, coronal MRI) pointing towards the contrast enhancement of the cervical carcinoma. The arrows in Picture C (ADC, coronal MRI) and D (DWI b=1000, coronal MRI) show restricted diffusion of the cervical carcinoma as well as the posterior wall of the urinary bladder with a low ADC value 0,58 × 10–3 mm2/s of the tumor. Picture E shows a coronal PET/CT with the green arrows pointing towards paraaortic and left iliac lymph node metastases with FDG accumulation. The orange arrow in picture E shows the FDG accumulation of the cervical carcinoma.

For comparison with rFIGO, cFIGO was available for 12/26 patients. In 17 % (2/12), rFIGO corresponded to cFIGO and in 83 % (10/12) staging differed with rFIGO compared to cFIGO. An example of upstaging with rFIGO is shown in Fig. 4.

There was minimal agreement between rFIGO and cFIGO (κ = 0.057), with low reliability (Cronbach’s Alpha 0.037), indicating poor internal consistency between rFIGO and cFIGO. Furthermore, two-way mixed ICC for single measures was 0.013 (95 % confidence interval –0.324 – 0.476) and for average measures 0.026 (95 % confidence interval –0.957 – 0.645) indicating negligible consistency, although not reaching statistical significance (p = 0.476).

### 3.3 Added value of rFIGO in terms of detection of metastatic disease

The frequency of metastatic disease based on rFIGO for the entire cohort was 19 % (5/26), of which 1/5 had pelvic lymph node metastases only and 4/5 had extra-pelvic metastases. Out of the 4 patients with extra-pelvic metastases, one had para-aortic lymph node metastases, two had tumour spread to adjacent organs, and one had tumour spread to distant organs.

### 3.4 Radiological vs. pathological FIGO staging

For comparison between rFIGO and pFIGO, data was available in 23/26 patients.

In 13 % (3/23), rFIGO corresponded to pFIGO and in 87 % (20/23) staging differed with rFIGO compared to pFIGO. The agreement was poor between rFIGO and pFIGO (κ = 0.169). Internal consistency was low to moderate with Cronbach’s Alpha (0.577) and also for individual assessments for single measures of two-way mixed ICC 0.314 (95 % confidence interval −0.057 – 0.625) and average measures (two-way mixed ICC 0.477 (95 % confidence interval −0.121 – 0.769), of which the latter two turned out statistically significant (p = 0.025).

### 3.5 Subtype analysis and staging discrepancies

Fig. 2 shows the distribution of UCC subtypes within our cohort. Among the patients with HPV+ SCC, 67 % (4/6) were assigned a higher stage by rFIGO compared to cFIGO. For the single patients with HPV-negative SCC and HPV status unknown SCC, both were upstaged by rFIGO. In the case of adenocarcinomas, 67 % (2/3) of the patients were assigned a higher stage with rFIGO.

## 4 Discussion

This retrospective study aimed to evaluate the added value of cross-sectional imaging techniques in staging of UCC with various histopathological subtypes by comparing staging with radiology (rFIGO) and clinical examination (cFIGO) using histopathology (pFIGO) as reference. Extended knowledge in this field may be of importance due to the expected shift in histopathological spectrum, and a deeper understanding of the discrepancies between cFIGO and rFIGO and their complementary nature may facilitate a tailored treatment approach for patients with different histopathological subtypes of UCC.

Our main result in this hypothesis-generating study was that rFIGO substantially upstages UCC compared to cFIGO, but without obvious differences in histopathological subtypes.

### 4.1 Clinical vs. radiological FIGO staging

The minimal agreement between clinical and radiological staging in our study underscores the challenges in achieving accurate staging in clinical settings, highlighting the supplementary diagnostic insight provided by rFIGO. Consistent with previous literature, our findings demonstrate that radiological staging frequently identifies more advanced disease stages compared to clinical evaluations alone [3], [11], [20], [21], [22]. While clinical staging is the first line in assessing the tumour stage, its inherent limitations, particularly detection of metastases, makes it insufficient as a stand-alone evaluation [23]. Treatment choices are dependent on the stage of UCC as well as on the presence of metastases at diagnosis and range from conization to chemoradiation [4]. The ability of radiological staging to detect more advanced stages of disease can therefore be pivotal in tailoring appropriate treatment strategies. The combination of radiological and clinical staging can bring complementary information and provide improved local tumour assessment and tumour resectability [15], [24].

### 4.2 Added value of rFIGO in terms of detection of metastatic disease

The importance of radiological imaging in enhancing staging decisions was particularly evident as it contributed to more accurate detection of disease extent, especially with whole-body imaging. In our study radiological evaluations led to the detection of clinically unknown metastases in 19 % (5/26) of our patients, including extra—pelvic metastasis in 15 % (4/26). These findings align with existing literature that emphasize the critical insight imaging provides in evaluating both the local and distant spread of tumours and in detecting lymph node metastases [14], [25], [26], [27].

Though every method of investigation has its advantages and disadvantages, complementing clinical examinations with conventional imaging modalities, can provide important details that could refine treatment strategies and improve patient outcomes, due to the inherent advantage of imaging being able to provide a whole-body assessment [15], [24].

### 4.3 Radiological vs. pathological FIGO staging

Although a significant minimal to moderate agreement was found between radiological and pathological data, it is important to note that even though not in agreement, 70 % of the patients (16/24) received a lower pFIGO stage compared to rFIGO. This emphasizes the usefulness of radiological staging, given that only a limited number of biopsies can be performed. For ethical reasons, not every lesion can be validated, and in some cases critical lesions are located in technically difficult areas, in proximity to large vessels etc., where the risk of the procedure is considered higher than the benefit.

### 4.4 Subtype analysis and staging discrepancies

In our cohort, MRI information led to a higher stage in 67 % (4/6) of HPV+ SCC cases, in 100 % (1/1) of patients with HPV-negative SCC, and in 100 % (1/1) of SCC where the HPV status was unknown. Adenocarcinomas also demonstrated changes in staging with rFIGO, with an increase observed in 67 % (2/3) of cases.

Even though current treatment is tailored according to tumour stage, the rising incidence of adenocarcinomas relative to SCC – due to the impact of screening and vaccination programs – necessitates a reassessment of diagnostic and therapeutic strategies [4], [8], [28]. While targeted therapy has emerged as an important approach in the treatment of various cancers, its application in UCC remains limited compared to other malignancies. However, ongoing research and clinical trials are exploring the potential of targeted therapies also for cervical cancers [28]. Considering the expected shift in histopathological subtypes, it is imperative to not only maintain stage-specific treatments but also to investigate targeted therapies tailored to these emerging subtypes, based on validated imaging-based staging [29]. This dual approach could enhance treatment efficacy and improve patient outcomes in the future.

### 4.5 Economic and practical implications

While radiological upstaging in UCC presents upfront costs, the possibility of tailored treatment could potentially minimize overtreatment and unnecessary complications and is expected to improve overall survival. Future studies should explore the cost-effectiveness of the implementation of routine radiological staging and its effect on different histopathological subgroups of UCC, considering the possible long-term reduction of unnecessary examinations and treatments, and subsequently improved survival rates [15], [30], [31], [32], [33].

### 4.6 Limitations

Our study’s robust findings are hampered by significant data limitations, due to its retrospective nature, with 54 % (14/26) of our patient cohort having incomplete or missing data. This limitation highlights the necessity for larger prospective studies designed to collect comprehensive and standardized data to avoid biases inherent in retrospective analyses. Additionally, multicentre studies could provide a broader data set, enhancing the generalizability of these findings and reducing variability in imaging interpretation.

### 4.7 Future research directions

Given the role of imaging in accurate staging of UCC, future research should focus on longitudinal prospective studies that evaluate the impact of radiology-based staging on treatment and survival outcomes. Moreover, technological advancements in imaging should continuously be explored. Such studies would provide deeper insights into the dynamic relationship between imaging accuracy and patient outcomes across different UCC subtypes.

## 5 Conclusion

In conclusion, rFIGO leads to substantial upstaging compared to cFIGO in primary staging of UCC, without obvious differences in histopathological subtypes. Although our sample size was too small to evaluate subtypes comprehensively, this aspect is interesting for future studies, especially considering the likely reduction in SCC HPV+ cases due to vaccination programs.

## Funding

This study was supported by internal funding from the Department of Diagnostics and Intervention, Umeå University. The sponsor was not involved in study design, collection, analysis and interpretation of data, writing of the report or in the decision to submit the article for publication.

## CRediT authorship contribution statement

**Stadler Carla:** Writing – review & editing, Writing – original draft, Visualization, Software, Investigation, Formal analysis. **Strandberg Sara N:** Writing – review & editing, Writing – original draft, Visualization, Validation, Supervision, Resources, Project administration, Methodology, Investigation, Funding acquisition, Formal analysis, Conceptualization.

## Declaration of Generative AI and AI-assisted technologies in the writing process

During the preparation of this work the authors used ChatGPT in order to improve readability and language. After using this tool, the authors reviewed and edited the content as needed and take full responsibility for the content of the publication.

## Declaration of Competing Interest

The authors declare that they have no known competing financial interests or personal relationships that could have appeared to influence the work reported in this paper.

## Data Availability

Anonymized data will be shared upon request.
